# Super High-Throughput Screening of Enzyme Variants by Spectral Graph Convolutional Neural Networks

**DOI:** 10.1021/acs.jctc.2c01227

**Published:** 2023-03-24

**Authors:** Carlos Ramírez-Palacios, Siewert J. Marrink

**Affiliations:** † Molecular Dynamics, Groningen Biomolecular Sciences and Biotechnology Institute (GBB), University of Groningen, Nijenborgh 7, 9747 AG Groningen, The Netherlands

## Abstract

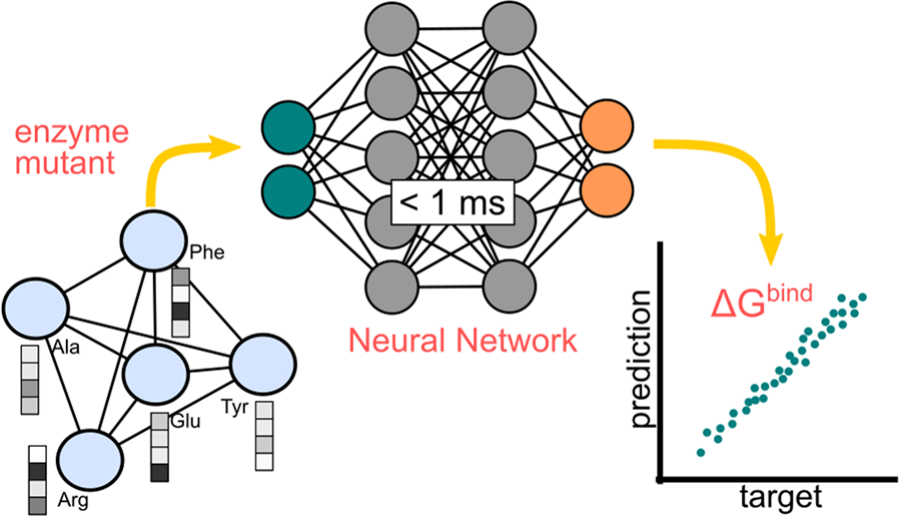

Finding new enzyme variants with the desired substrate scope requires screening through a large number of potential variants. In a typical *in silico* enzyme engineering workflow, it is possible to scan a few thousands of variants, and gather several candidates for further screening or experimental verification. In this work, we show that a Graph Convolutional Neural Network (GCN) can be trained to predict the binding energy of combinatorial libraries of enzyme complexes using only sequence information. The GCN model uses a stack of message-passing and graph pooling layers to extract information from the protein input graph and yield a prediction. The GCN model is agnostic to the identity of the ligand, which is kept constant within the mutant libraries. Using a miniscule subset of the total combinatorial space (20^4^–20^8^ mutants) as training data, the proposed GCN model achieves a high accuracy in predicting the binding energy of unseen variants. The network’s accuracy was further improved by injecting feature embeddings obtained from a language module pretrained on 10 million protein sequences. Since no structural information is needed to evaluate new variants, the deep learning algorithm is capable of scoring an enzyme variant in under 1 ms, allowing the search of billions of candidates on a single GPU.

## Introduction

Enzyme engineering is the process of tailoring existing enzymes to enhance some property through alteration of the sequence of amino acids that constitute the enzyme.^1^ The property of interest is typically—but not limited to—catalytic activity, substrate specificity, enantioselectivity, or thermostability. The most well-established strategies for tailoring enzymatic properties are directed evolution^2^ and rational design.^3^ The two strategies require large amounts of experimental or computational effort to find suitable variants.

A third possibility to guide enzyme engineering efforts is through machine learning (ML), and in particular deep learning (DL) approaches. Two of the aspects that have hampered the application of ML in protein engineering are the large data sets needed for training ML models and the challenge of meaningfully representing molecules. The former has been addressed by more efficient architectures (e.g., few-shot or one-shot learning), by designing unsupervised learning tasks, or by using the data generated by classical molecular modeling methodologies (e.g., molecular dynamics trajectories) as training data. The latter aspect has been addressed by representing molecules as graphs,^4^ sequences,^5,6^ Cartesian coordinates,^7^ or vectors obtained via representation learning.^8^ ML-assisted protein engineering is thus an emerging field, with recent successes including classification and prediction of the location of the binding site,^9^ prediction of the protein 3D structure from the sequence,^10^ design of artificial enzymes,^11^ de novo protein design,^12^ scoring protein–protein docking complexes,^13^ scoring drug target interactions,^4,14^ low-data drug discovery with one-shot learning,^15^ predicting the native-like probability of every amino acid in a protein sequence,^16^ and protein thermostability.^17^

High-throughput screening of enzyme variants can be done experimentally or *in silico*. In wet-lab experiments, it is possible to screen >10^6^ variants per hour.^18^ The number of variants that can be screened *in silico* depends on the specific methodology used, which determines the accuracy, and on the computational resources available. In either case, screening the entire combinatorial space may not be the most cost-effective strategy to find suitable candidate variants. A good alternative would be to train some algorithm, for example a neural network, that learns to combine mutations and predict the outcome at lightning-fast speed (super high-throughput screening). DL-assisted super high-throughput screening of enzyme variants has been attempted before with varying degree of success.^19^ Cadet et al.^20^ used a ML approach with protein spectra obtained via Fourier transform of the protein sequence to predict the enantioselectivity of variants of epoxide hydrolase. Their ML approach can evaluate 2^30^ enzyme variants in less than 48 h (∼0.16 ms per variant). Wu et al.^21^ used shallow neural networks to evaluate combinatorial libraries from human GB1 binding protein (fitness) and a putative nitric oxide dioxygenase from *Rhodothermus marinus* (enantioselectivity). Liao et al.^22^ tested the ability of eight ML algorithms to evaluate mutants of proteinase K using a small data set (<100 variants) with experimentally determined enzymatic activities.

The approach presented in this study uses deep Graph Convolutional Neural Networks (GCNs) to explore the fitness landscape of enzyme variants trained on large data sets (10^4^ variants) generated by traditional molecular modeling approaches, enabling the evaluation of 10^8^ variants in less than 24 h.

The enzyme herewith explored is an ω-transaminase from *Vibrio fluvialis* (*Vf*-TA) that catalyzes the conversion of ketones into chiral amines with high enantioselectivity.^23^*Vf*-TA is interesting from a protein engineering perspective for industrial production of chiral amines.^24^ However, the substrate range of *Vf*-TA, and other ω-transaminases, often needs to be tailored to produce the amine of interest.^25,26^ Searching for enzyme variants that are better suited to accommodate the molecule of interest is a laborious task that can be aided by computational design approaches. Rational design of new *Vf*-TA variants can be performed *in silico*, in which new variants are generated and scored computationally, and only the best variants are selected for experimental verification. A computationally inexpensive strategy for scoring the goodness of *Vf*-TA variants is by measuring the Rosetta Interface Energy of the enzyme-ligand intermediate complex. The Rosetta Interface Energy (from now on referred to as the binding energy) has been shown to correlate well with the enzymatic activity of ω-transaminases.^27,28^ Libraries of Rosetta-generated mutants labeled with the binding energy (*y*_*i*_) were used to train NNs to predict the binding energy (*ŷ*_*i*_) of unseen variants.

GCNs extend the convolution from regular (e.g., images) to irregular (graphs) representations.^29,30^ Similar to classical Convolutional Neural Networks (CNNs), the pooling operation allows GCNs to learn increasingly abstract representations and to discard unnecessary information. However, the non-Euclidean nature of graphs makes the convolution operation difficult to define and the application of GCNs has lagged behind respect to CNNs. GCNs have been used before to process graphs representing proteins to score protein–protein^13,31^ or protein–ligand^4^ complexes. The two main approaches to handling convolutions with irregular shapes are spatial and spectral convolution. The former attempts to perform convolution directly in the vertex domain typically by allowing information to flow between neighboring nodes through architecture-specific propagation rules. The latter attempts to perform convolutions in the frequency domain (Fourier-like basis), centering its attention in the graph Laplacian matrix, ***L*** = ***D*** – ***A***, where ***D*** is the diagonal degree matrix, , and ***A*** is the adjacency matrix.

In this work, the spectral GCN proposed by Bianchi et al.^32^ is used to analyze the *Vf*-TA variants. The architecture was chosen because the pooling operation clusters the nodes not just according to graph topology but also to node features, which is useful if the graph topology does not change across mutants but the node features do.

## Methods

### Training Data Sets

**Figure 1:**
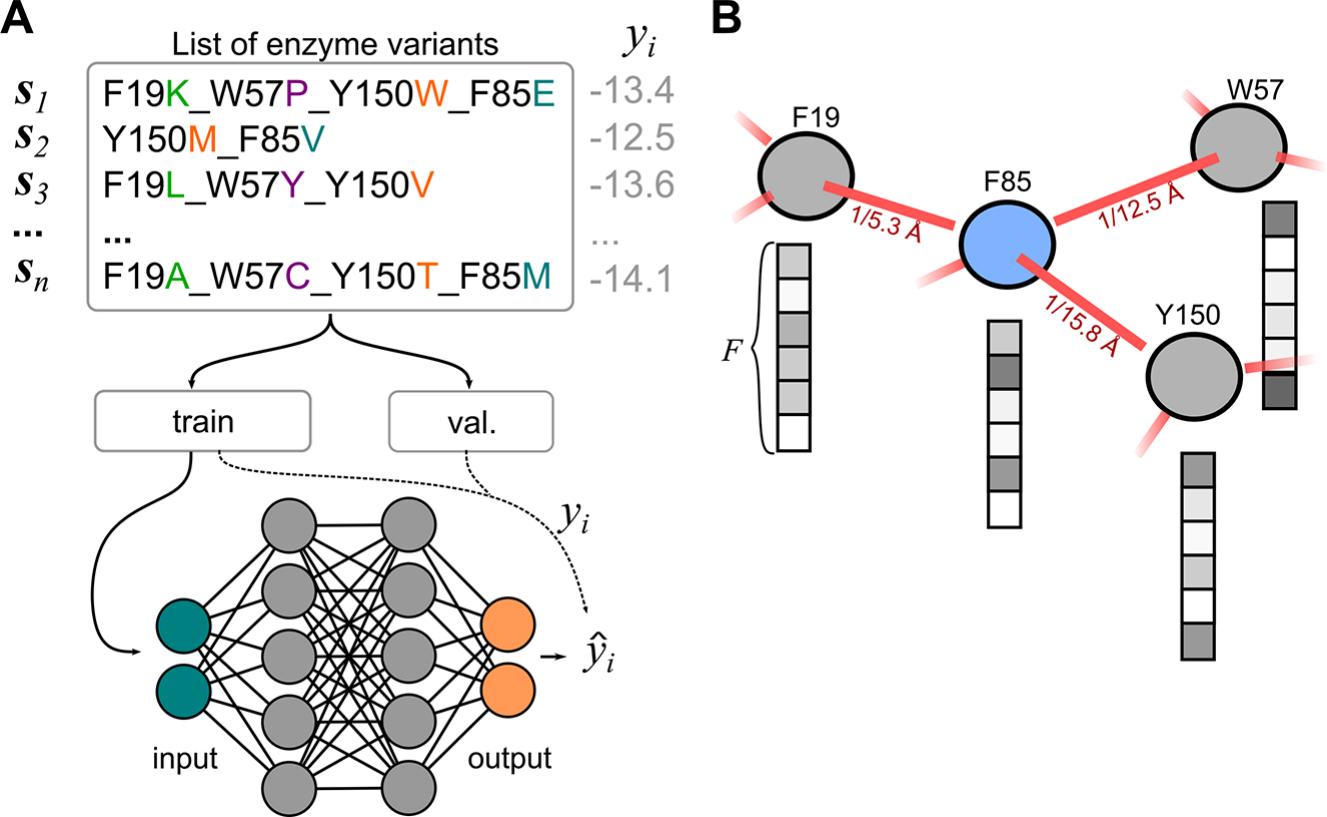
A) Proposed methodology. In the data set, the enzyme variants (***s***_*i*_) are labeled with the binding energy of the variant in complex with the ligand (*y*_*i*_). The data set is used to train a NN, which learns the synergic relationships between mutations and can make predictions for unseen mutants. B) Graph representation of the query variant. Nodes (circles) represent the protein residues connected by edges (red lines). The nodes are characterized by a set of attributes (checked rectangles) representing physicochemical properties of the amino acid.

A randomly sampled library containing 10,000 *Vf*-TA variants was created to be used as training data. The variants were created by randomly mutating a set of predetermined *N*_*hot*_ hotspots, to generate *L*_th_-order mutants (*L* = 1, single mutant; *L* = 2, double mutant; etc.), into one amino acid sampled from the set of 20 naturally occurring amino acids, *AA* = {*A,C,D,E,F,G,H,I,K,L,M,N,P,Q,R,S,T,V,W,Y*}. Each example, ***s***_*i*_, was labeled (*y*_*i*_) by calculating the binding energy between the enzyme variant and the ligand (Figure 1A). The ligand (Figure S1) was maintained constant within the data set of 10,000 variants. The full data set was split into training (80%), validation (10%), and test (10%) subsets. The standard data set referred to in the paper is . Additional data sets were generated by changing the number of hotspots allowed to mutate *N*_*hot*_ = {4,6,8}, the maximum *L*_th_-order of the generated mutants, the number of amino acids allowed as target mutations |*AA*| = {10, 20}, or the identity of the ligand LIG = {**E1**, **E2**, **E3**, **E4**, **E5**}.

### Data Labeling

The enzyme variants were labeled using the binding energy of the query variant in complex with a ligand. The binding energy is the Rosetta Interface Energy and, unless otherwise indicated, the ligand is **E4** (Figure S1). The procedure for performing molecular docking and measuring binding energies is the same as described in Ramírez-Palacios et al.^33^ Very briefly, the external aldimine form of the query compound is docked into the binding site of *Vf*-TA and the binding energy is calculated and averaged over 10 replicas. The binding energies were calculated using the Rosetta suite (build number 57927).^34^ The binding energies were whitened to have zero mean and standard deviation of one, *y* = (*x* – *mean*)/*stdev*.

### Graph Representation of the Protein

The enzyme variants were represented as graphs formed by protein residues (nodes) with pairwise connections (edges). Let  be a graph, where  is the set of nodes with F-dimensional real attributes , and ***ε*** is the set of edges with S-dimensional real attributes  connecting nodes *i* and *j*. For computation, the graph  is represented by a binary adjacency matrix , node features , and edge features , where *N* is the number of nodes (i.e., the number of protein residues).^35^

**Figure 2:**
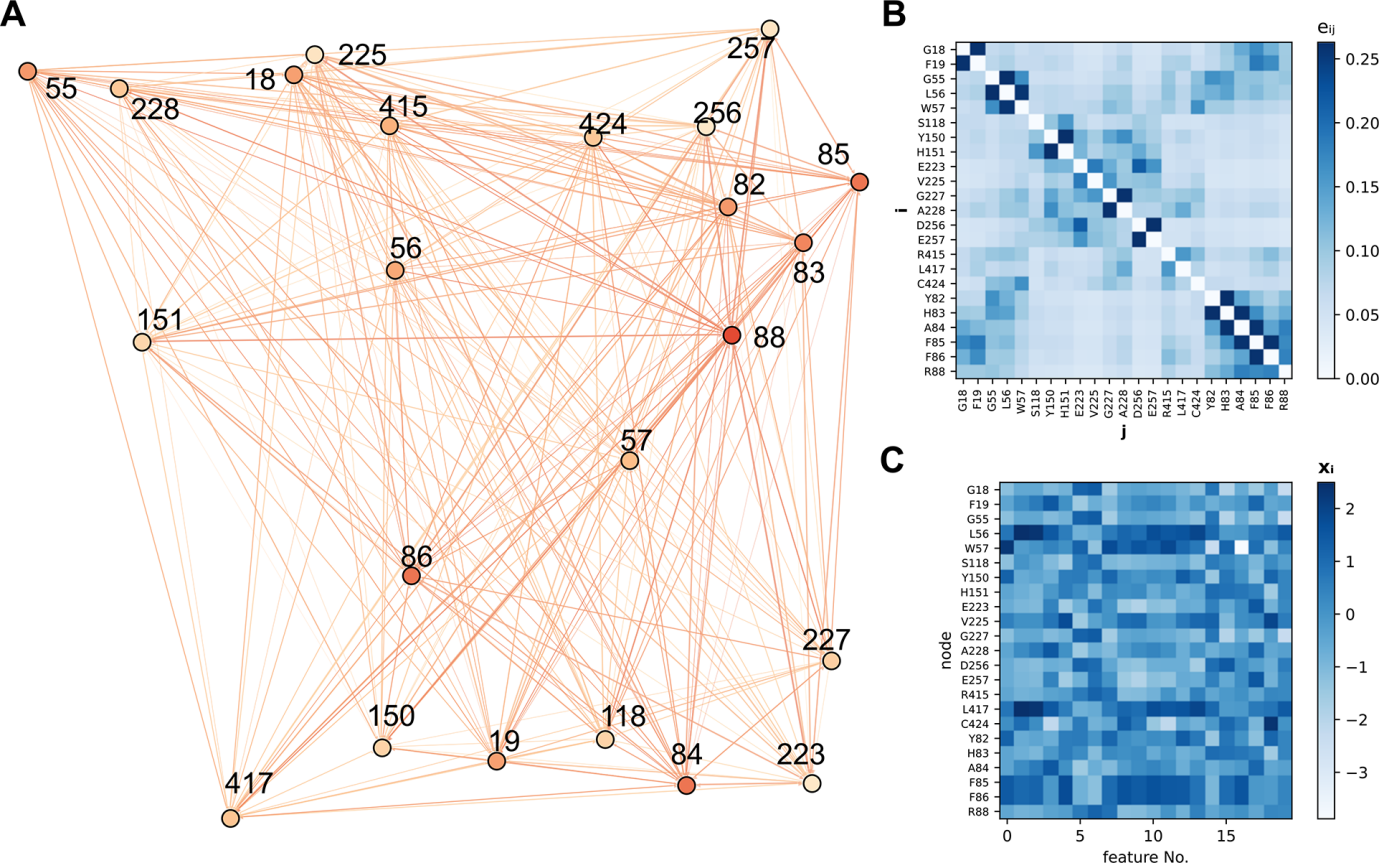
A) Example of a graph representation of the binding site of *Vf*-TA. B) Heatmap showing the edge weight matrix, where the weight of each edge (*e*_*ij*_) is the inverse pairwise distance between nodes *i* and *j*. The edge features were maintained constant across mutants. C) Heatmap showing the feature matrix, where each node (*rows*) is described by features (along the *columns*) which are the physicochemical properties of the constituent amino acid.

The graph representation of *Vf*-TA included only nodes representing residues near the binding site (*N* = 23) (Figure 2A), and all other residues were truncated away. Including only a small subset of protein residues to form the graph helps ease computations. A smaller subset of those 23 residues was allowed to mutate, *N*_*hot*_ ∈ *N*|_*N*_*hot*_ = {4,6,8}_. Featurization of the node matrix, ***X***, was carried out using *F* features taken from the AAindex^36^ (Figure 2C and S4). The AAindex is a collection of 529 biophysical and biochemical properties of amino acids,. All nodes in the graph were connected to all other nodes. Finally, the edge attributes, ***E***, were defined as the inverse of the pairwise distance between the protein residues C_α_ atoms, *e*_*ij*_ = 1/∥***e***_*i*_ – ***e***_*j*_∥_2_. Since the position of the protein C_α_ atoms does not change significantly upon mutagenesis, the edge tensor, ***E***, was set to be invariant to the identity of the mutant (Figure S2). The main advantage of keeping the edges constant across mutants is that evaluation of new variants can be done from the protein sequence alone, which massively reduces the computational cost.

### Graph Convolutions

The input graph, , goes through a stack of convolutional layers to generate an increasingly more abstract representation of the input signal. The graph convolution layers used in this work, proposed by Bianchi et al.,^32^ were obtained from the *Spektral* python library, and consist of a message-passing followed by a graph pooling layer.

**Figure 3:**
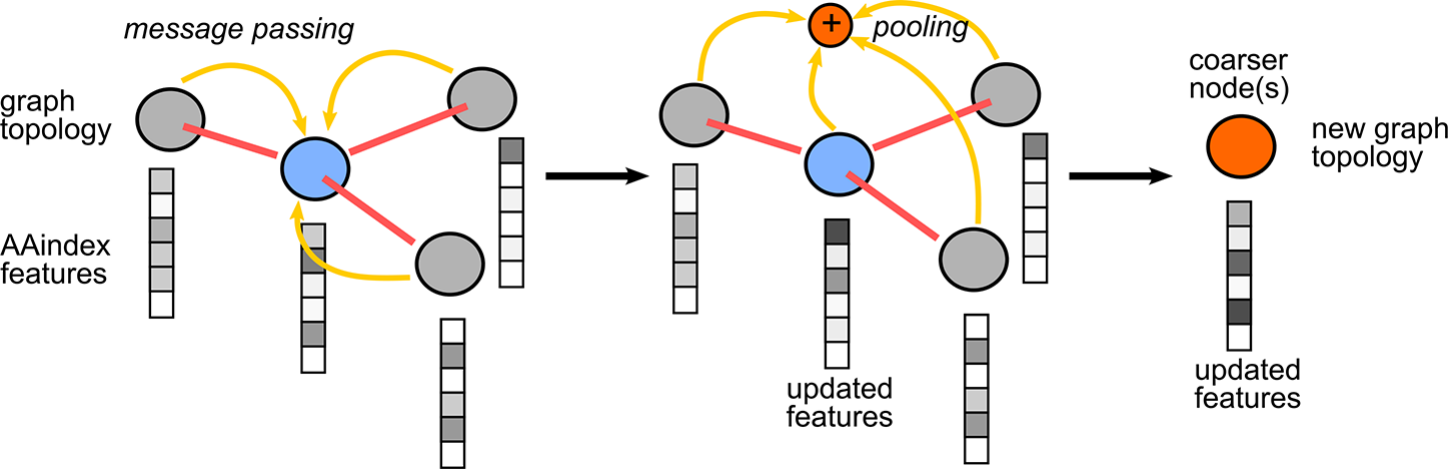
Spectral graph convolution as implemented by the employed layers. The message-passing operation consists of neighboring nodes passing information to each other, which also depends on edges. The pooling operation consists of clustering a subgroup of nodes together according to graph topology and node features. The purpose of these operations is to reduce complexity and yield a more abstract representation. The coarser node(s) (*orange*) contains information from all parent nodes (*gray* and *blue*).

The message passing operation is performed using the GCSConv layer. In this operation, the graph topology remains intact while the features of each node are updated by rules learned through training (Figure 3). The output of the message-passing layer is
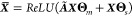
1Where ***Θ***_***m***_ and ***Θ***_***s***_ are the trainable weights of the mixing and skip component, respectively. ***Ã*** is the symmetrically normalized adjacency matrix, ***Ã*** = ***D***^–1/2^***AD***^–1/2^.

The pooling operation is performed using the MinCutPool layer on ***X̅***, which uses a skip connection to ***Ã*** and passes the input through an MLP, to cluster the graph and yield the cluster assignment matrix ***S***. Graph clustering helps discard superfluous information, provides translation invariance, and reduces model complexity. The coarsened graph, (***A***^*pool*^, ***X***^*pool*^), is thus a more abstract representation of the protein. The pooling operation clusters the nodes according to both graph topology and node features (nodes with similar features are more likely to be clustered together).

The message-passing and clustering operations can be performed several times on the newly generated graphs. At the end, the graph is linearized and passed through a single FC layer with one output unit.
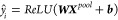
2Predictions can then be evaluated using the Mean Squared Error (MSE).
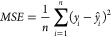
3

### Model Build and Training

The graph convolutional network models were run using the *Spektral*(35) and *TensorFlow*(37) 2.4 libraries. The model is composed of an input layer, a GCSConv layer, a maxpooling layer, a MLP layer, and an output layer.^32^ The models were trained for 200 epochs using the ADAM optimizer with a learning rate of 1 × 10^–4^ and parameters β_1_ = 0.9, β_2_ = 0.999, and ε̂ = 1 × 10^–7^.^38^ The input data was fed to the network in batches of size 1 for training. To avoid overfitting, early stopping with patience of 40 was used.

### Representation Learning Using a Pretrained LSTM Model

**Figure 4:**
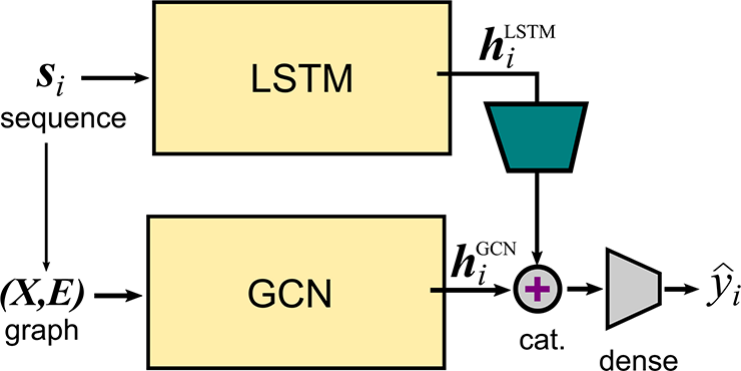
Representation learning approach. The query sequence is mapped into a latent space representation (***s***_*i*_→***h***_*i*_^*LSTM*^) by a pretrained LSTM network. The same protein sequence can be represented as a graph (***G***_*i*_ = (**X**,**E**)) and passed through a pretrained GCN network (***G***_*i*_→***h***_*i*_^*GCN*^). The two latent space representations are then concatenated (cat.) and passed through a shallow network to yield a prediction. The trapezoid represents the dimensionality reduction via either an FC or a CNN module.

To further improve the accuracy of the trained GCN models, protein sequence embeddings were fed to the NN (Figure 4). The sequence of the query variant, , is converted to a latent space representation, , by a pretrained bidirectional LSTM model. The LSTM model was trained with ∼10 million protein sequences from the Pfam database (https://github.com/flatironinstitute/DeepFRI). The latent space representation is then reshaped to a low-*d* vector by passing it through a 2D-CNN module that treats the vector embedding as an image. The hidden representation passes through two convolutional and two pooling layers to yield an 8-*d* vector. The 8-*d* vector is then concatenated to the output of the penultimate layer of the already-trained GCN module. The resulting 16-*d* vector is finally passed through a single dense layer to produce the final 1-*d* output vector, .

## Results

### The Trained Spectral Graph Convolutional Network Can Predict Binding Energies with High Accuracy

**Figure 5:**
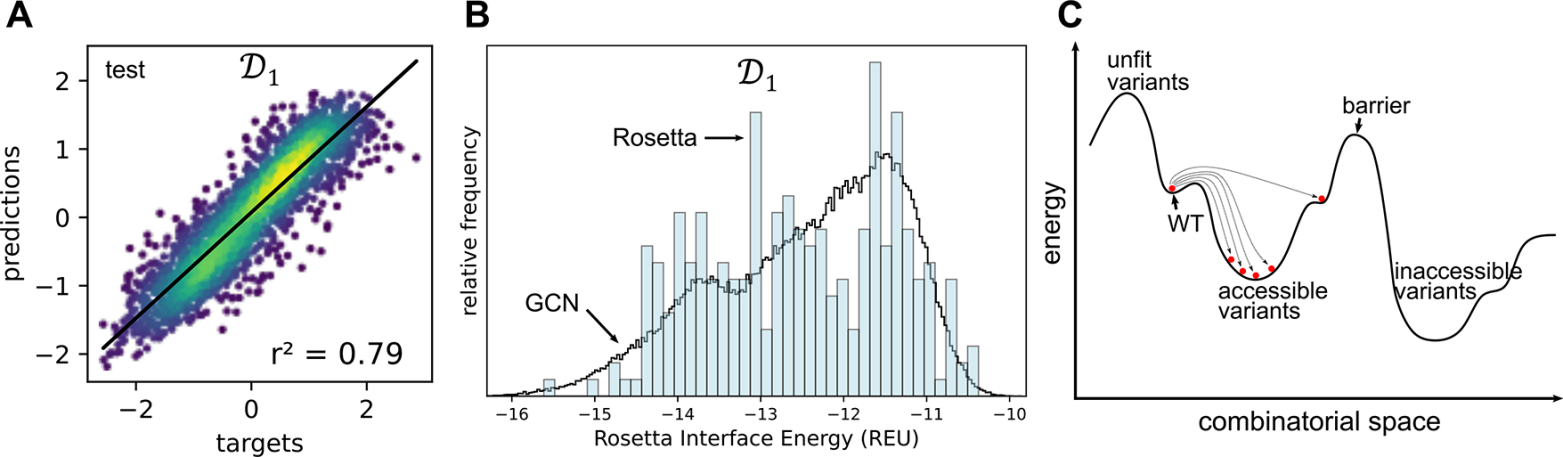
A) KDE scatter plot showing the correlation between the GCN-predicted binding energies (*y-axis*; *ŷ*_*i*_) and the Rosetta scores (*x*-axis; *y*_*i*_) with the test data set (*n* = 2,000). B) Histogram showing the distribution of binding energies from the entire combinatorial space (black line) obtained with the trai*n*ed model (*n* = 160,000), overlapped to the histogram obtained with designs proposed by Rosetta (not used for training) (blue bars) (*n* = 800). C) When Rosetta is tasked with proposing new variants from a set of predefined mutable positions, the generated variants will tend to be nearby in combinatorial and conformational space. The variants easily accessible are located close to the wild-type enzyme. The variants that are farther away are less likely to be generated, especially if a barrier needs to be overcome.

A neural network was trained on a mutant library of *Vf*-TA enzyme-ligand complexes generated and scored by Rosetta. The data set is a collection of variants, in which a fixed number of positions (*N*_*hot*_ ∈ {4,6,8}) were randomly mutagenized. The targets (*y*_*i*_) correspond to Rosetta binding energies, but any other type of binding energy would work. The network is unaware of the identity of the ligand used for training, and predictions (*ŷ*_*i*_) are based solely on the protein sequence (***s***_*i*_). The protein sequence is converted to a graph representation of the binding site, , to serve as input for the GCN. The neural network thus predicts the binding energies of unseen enzyme variants using the graph representation of the protein (Figure 2A). The trained model was not only able to predict with high accuracy the binding energies of unseen variants (Figure 5A and S3), but also does it 6 orders of magnitude faster than Rosetta. Evaluation takes approximately 1.36 ms per screened variant on a single GPU, but it can be parallelized with more workers within the same GPU. This massive speed-up opens the possibility of scanning billions of enzyme variants at a low computational cost. For example, the search space of all eighth-order variants (octuple mutants) is 20^8^ = 2.56 × 10^10^, which is virtually inaccessible by traditional methods. The proposed methodology enables screening of the entire search space (Figure 5B).

The GCN model trained on  was used to screen the whole combinatorial space of , i.e., 160,000 mutants (Figure 5B *black line*). The distribution of the entire combinatorial space of  was then compared to the distribution of mutants obtained when running Rosetta to propose new mutants (*n* = 2,000). In the run, Rosetta is tasked with proposing mutants (Figure 5B *blue bars*) where positions 19, 57, 150, and 85 are mutated, without limitations or constraints as to the identity of the amino acids to which these four positions are allowed to mutate. The two distributions are similar, which suggests that the GCN model is able by brute force to propose as good mutants as Rosetta can but at a lower computational cost. Moreover, the brute force approach can explore variants that are not near the initial variant (wild-type) in combinatorial or conformational space (Figure 5C). The screening of the entire combinatorial space of  by the trained GCN predicted the following five mutants to be the best: F85A_W57F_Y150F (−15.14), F85P_W57F_F19Y (−15.15), F85 V_W57F_Y150F (−15.18), F85G_Y150W_F19W (−15.19), and F85P_W57F_Y150F_F19Y (−15.29). The number in parentheses is the energy predicted by the GCN model. Rosetta later scored these mutants with energies of −15.28, −15.04, −15.12, −14.35, and −15.52 kcal/mol, respectively. Except for the mutant with a score of −14.35, the other four mutants seem promising candidates. For reference, the training data set contained mutants in the range of −8.84 to −15.38. How far the trained model can extrapolate to energies outside the range of the training data set is addressed later in the text and Figure S7.

The main ligand used in this study is the external aldimine intermediate of compound 4 (Figure S1). In addition to **E4**, other ligands were used for training and testing: **E1**, **E2**, **E3**, and **E5**. The ligands **E1**–**E5** were chosen because it has been experimentally shown that the Rosetta score is a good predictor of the enzyme’s activity toward these five compounds.^27^ Overall, the trained models are accurate in making binding energy predictions with all five ligands (Figure S6).

The proposed architecture is ideal for the task because the MinCutPool layer assigns clusters based on both graph topology (edge attributes) and node features.^32^ Since the majority of the residues in the protein are never mutated, the graph topology is constant across variants. Because of that, clustering methods that heavily rely on graph topology would not perform ideally. For reference, a model using the classical architecture of Kipf and Welling^39^ (*Spektral* layer: GCNConv) was trained and the results show that while the model is capable of scoring new protein variants, its performance is inferior to GCSConv (Table 1). In general, the power of the graph representations is clear from the poor performance of a FC dense network, in which no learning occurred.

**Table 1:** Results of the Trained Models on the Validation and Test Data Sets for Various Architectures^a^

	Validation			Test		
Model	↑Acc.	↓Loss	↑r^2^	↑Acc.	↓Loss	↑r^2^
GCSConv	0.44	0.23	0.76	0.46	0.23	0.77
GCSConv (no edges)^b^	0.60	0.56	0.44	0.60	0.54	0.47
GCSConv (extra nodes)^c^	0.75	0.72	0.31	0.75	0.66	0.34
GCNConv	0.59	0.46	0.57	0.59	0.45	0.55
Dense	0.00	1.00	–1.0	0.00	1.00	–1.00

a Data Set is .

b All edges were simply set to 1.

c Number of nodes was increased from 23 to 291. Evaluation time increases to 14.1 ms per variant.

Since by definition the edge matrix is the same across mutants (Figure S2), one could ask whether the pairwise distances are adding any useful information to the network. Thus, a model was trained without edge information (the weights of all edges were simply set to 1, *e*_*ij*_ = 1), which resulted in a performance penalty (Table 1). This means that the extra information that the edge matrix provides to the network, i.e., the relative positions of the protein residues, does indeed improve performance. Additionally, one could ask whether the number of residues (nodes) selected beforehand to be part of the graph representation is optimal. After all, only 4–8 residues are allowed to mutate, and the other 15–19 residues are kept constant. The network may benefit from knowing the identities of more residues as this would provide additional information on the environment of the binding site. A model trained with 291 nodes did not improve performance, and on the contrary increased the evaluation time 10-fold and reduced the accuracy of the predictions (Table 1).

The data set insofar used for training and testing the NNs contained single, double, triple, and quadruple mutants (*L* = 1, 2, 3, and 4, respectively) on *N*_*hot*_ = 4 positions (positions 19, 57, 150, and 85 are allowed to mutate). With *N*_*hot*_ = 4, the total combinatorial space is 1.6 × 10^5^ variants, which means that the training data set of 1 × 10^4^ variants covered 6.25% of the total combinatorial space (Figure S5). Additional data sets with more hotspot positions and higher-order mutants were tested (Table 2). With *N*_*hot*_ = 6, the training data represents 0.015% of the total combinatorial space of 6.4 × 10^7^ variants. And with *N*_*hot*_ = 8, the training data represents a mere 0.000039% of the total search space of 2.56 × 10^10^ variants. Gratifyingly, the GCN models were still able to perform well on the prediction task but with a reduced accuracy (r^2^ = 0.52 for *N*_*hot*_ = 8) (Table 3). An easy solution to increase the accuracy of the trained NN is to increase the number of examples in the training data set. Training the NN with a larger data set of 5 × 10^4^ variants (0.00019% of the total combinatorial space) marginally increased the accuracy to r^2^ = 0.59. However, increasing the size of the training data set is not always the optimal solution, especially when more sophisticated and expensive methodologies are used to label the mutants. Instead, we increased accuracy by feeding vector embeddings from a pretrained language model (see below).

**Table 2:** Data Sets Used for Training^a^

Name	*N*_*hot*_	*N*_*hot*_ positions	*L*_*max*_^b^	*|AA|*^c^	Search space^d^
D1	4	F19,W57,Y150,F85′	4	20	1.6 × 10^5^
D2	6	F19,W57,Y150,A228,R415,F85′	6	20	6.4 × 10^7^
D3	8	F19,W57,Y150,V225,A228,R415,F85′,F86′	8	20	2.56 × 10^10^
D4	8	F19,W57,Y150,V225,A228,R415,F85′,F86′	4	20	1.12 × 10^7^
D5	4	F19,W57,Y150,F85′	4	10	1 × 10^4^

a The number of examples per data set is 10,000 variants.  is the main data set used in this study, it contains mutants of degrees 1, 2, 3, and 4 (*L*_max_ = 4) in 4 positions (*N*_*hot*_ = 4) that are allowed to mutate to any of the 20 standard amino acids (|*AA*| = 20). The search space of  is therefore 1.6 × 10^5^. Data set  (*L*_max_ = 8, *N*_hot_ = 8) also resembles conditions relevant in enzyme design campaigns, with a search space of 2.56 × 10^10^. In all cases, ligand is **E4**.

b Maximum allowed mutant degree. *L*_*max*_ = 4, means that single (*L* = 1), double (*L* = 2), triple (*L* = 3), and quadruple (*L* = 4) mutants were allowed.

c The number of amino acids allowed as target mutation, |*AA*|, was reduced to 10 in : *AA* = {*A,C,D,E,G,H,I,K,L,M*}, to see if the trained model could generalize to unseen amino acids, i.e., *AA* = {*F,N,P,Q,R,S,T,V,W,Y*}.

d The search space for each data set was calculated with the following formula: *C*(*N*_*hot*_*,L*)·|*AA*|^*L*^, where *C*(*N*_*hot*_*,L*) is the combination of *L* items (mutant degree) taken from the set of size *N*_*hot*_ (number of hotspots), and |*AA*| is the number of amino acids allowed (normally 20).

**Table 3:** Performance of the GCN and LM-GCN Models on Various Data Sets^a^

		GCN model				LM-GCN model			
Data set		Train		Validation		Train		Validation	
Train	Eval.	↑r^2^	↓loss	↑r^2^	↓loss	↑r^2^	↓loss	↑r^2^	↓loss
D1	D1	0.819	0.182	0.813	0.188	0.808	0.158	0.805	0.194
D2	D2	0.574	0.427	0.552	0.435	0.667	0.310	0.651	0.356
D3	D3	0.502	0.498	0.497	0.489	0.642	0.347	0.625	0.368
D4	D4	0.596	0.405	0.588	0.405	0.601	0.399	0.597	0.405
D5	D5	0.786	0.213	0.778	0.227	0.852	0.152	0.855	0.147
D5	D1	–	–	0.602	0.403	–	–	0.638	0.361
D4	D3	–	–	0.396	0.587	–	–	0.449	0.542

a The architecture of the LM-GCN model is shown in Figure 6. The size of the data sets is 10,000 examples, with 80:20 split for training and validation, respectively.

Unsurprisingly, the degree of the mutants in the training data set had an impact on the model’s performance. A model trained and evaluated on a data set containing first–fourth order mutants (e.g., , r^2^ = 0.588, loss = 0.405) performed better than a model trained and evaluated on a data set containing first–eighth order mutants (e.g., , r^2^ = 0.497, loss = 0.489). Models trained with lower-order mutants did not generalize well to higher-order mutants, as shown by the results of the model trained on  but evaluated on  (r^2^ = 0.396 and loss = 0.587) (Table 3). Furthermore, the NN did not generalize to unseen amino acids either. A NN trained on the  data set, which contained mutants with only 10 amino acids (Ala, Cys, Asp, Glu, Gly, His, Ile, Lys, Leu, or Met), was not particularly good at evaluating mutants from the  data set, which contained mutants with all 20 amino acids (r^2^ = 0.602, loss = 0.403) (Table 3).

### Vector Embeddings from a Pretrained Bidirectional LSTM Module Can Increase Accuracy

The pretrained LSTM model maps the protein sequence, ***s***_*i*_, to a vector representation, ***h***_*i*_, that can be fed to the GCN model to improve predictions. The projection, ***s***_*i*_→***h***_*i*_, is made in the context of all the 10 million Pfam protein sequences with which the LSTM model was trained. Thereby, mutant sequences that are close to the sequences with which the LSTM was trained (e.g., mutant sequences that do not have a Pro residue within an α helix region) will be close in the embedding space. The vector embeddings, , are passed through a 2D-CNN module to project them to a low dimensional 8-*d* vector. The 8-*d* vector is concatenated with the 8-*d* vector produced by the trained GCN module, resulting in a 16-*d* vector that can then be passed through as single dense layer with one output unit to generate a prediction, *ŷ*_*i*_. The model presented in Figure 6 was trained keeping both the GCN and the pretrained LSTM modules frozen. After training for 200 epochs, the resulting LM-GCN model outputted better predictions than the GCN model alone (Figure 6B,C; Table 3). Originally, a representation learning strategy more similar to the one reported by Gligorijevic et al.^9^ was attempted, using the hidden state representation from the last amino acid position of the input sequence of length |***s***_*i*_| = 452. The mutations in the *Vf*-TA data set occur in positions 19, 57, 85, 86, 150, 255, 228, and 415, which means that the signal from the early substitutions (e.g., position 19) will have degraded by the time we arrive at the last position (Figure S8). Therefore, the embeddings coming from all positions were used, more similar to the strategy of Bepler and Berger.^40^

**Figure 6:**
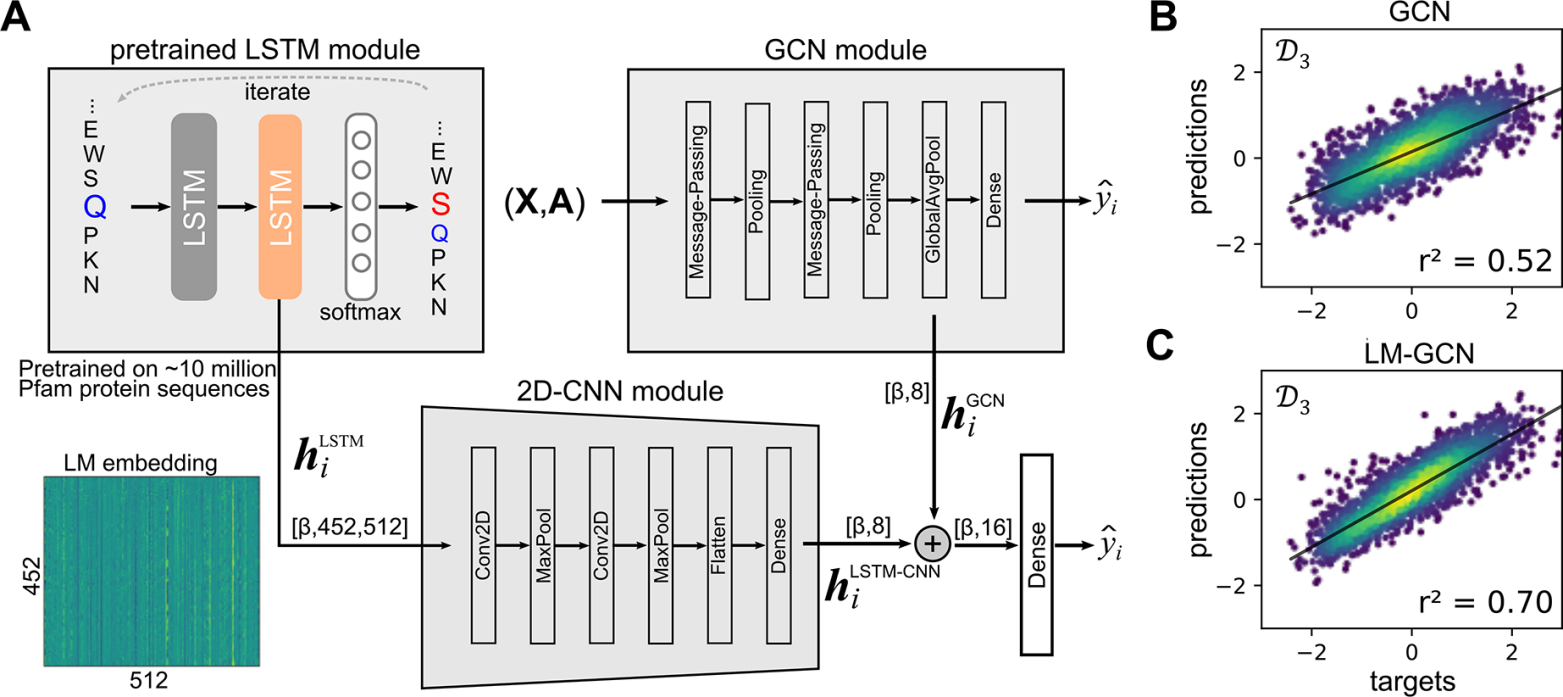
LM-GCN model. A) The LM-GCN model consists of a pretrained LM module, a trained GCN module, and a 2D-CNN module. All layers from the LM and GCN modules are frozen when training the LM-GCN model during 200 epochs. The prediction reached by the combined LM-GCN model is better than the predictions of the GCN model alone. β represents the batch size. B) and C) are the correlations for the GCN and LM-GCN model, respectively. Training was done on the *N*_*hot*_ = 8 data set. The 2D-CNN module using the LM embeddings (, where 452 is the length of the enzyme sequence and 512 is the number of units in the pretrained LM) as input vectors can achieve a correlation r^2^ = 0.43 on its own, lower than the correlation of the GCN and LM-GCN models.

### Applicability in Protein Engineering Campaigns

There are three limitations to consider for the application of the presented methodology in protein engineering campaigns. 1) Generation of the training data set can be expensive. Scoring 10,000 mutants using Rosetta with 10 replicas each required 1,600 core-hours, which is equivalent to a runtime of 133 h on a desktop with 12 threads per CPU (HP workstation Z4 with an Intel Xeon W-2135 processor). Training the model only takes 0.5–2 h on a GPU (NVIDIA GTX 1080). Once the model has been trained, evaluation of new mutants takes less than 1 ms, which is 1 × 10^6^ times faster than Rosetta. 2) If *good mutants* are not already present in the training data set, the model is not expected to extrapolate to the region of good scores. However, we did find that a model trained only with bad mutants was still able to give the best scores it could to good mutants when tested (Figure S7). 3) Mutants that significantly change the protein backbone conformation might prove difficult because we set the edge attributes, i.e., the pairwise distances between protein residues C_α_ atoms, to remain constant across mutants. In practice, we do not expect the protein backbone to change considerably with mutants of degree 1–8 (Figure S2) or Rosetta to be able to move the protein backbone to beyond ∼1 Å.^41^ Moreover, the NN was observed to still make predictions even when all edge attributes were set to 1.0 (Table 1), which means that while edge attributes help accuracy, they are not essential. While these limitations should generally not hinder applicability to protein engineering campaigns, we believe the importance of this study should be in the fact that a simple algorithm lacking much awareness about the identity of the molecules involved is capable of learning the intricate synergic relationships needed to assess new combinations of mutants.

## Discussion

In this work, it has been shown that it is possible to train a neural network to learn the intricate synergic relationships needed to assess mutants resulting of the combination of two or more individual mutations. The main advantage of the presented deep learning strategy for predicting binding energies of protein–ligand complexes is the massive speed-up compared to traditional computational HTS screening methods. The trained DL model can, after ∼2 h of training, predict the binding energy of unseen enzyme variants in 1.36 ms with high accuracy, enabling super high-throughput screening. Computational strategies for fast screening of enzyme variants are useful because of the astronomic size of the combinatorial space that can be explored. Targeted mutagenesis efforts explore a limited number of variants in search of a good variant, typically by mutating 4–8 positions around the binding site, but the combinatorial space of 8 positions amounts to 20^8^ possible variants. One possibility could be to generate all possible single mutants in 8 positions (8 × 20 = 160 variants) and combine the best amino acid from each position to generate double or higher-order mutants. However, such a strategy would not take correlations into account, i.e., the effect that mutating a position in the vicinity can have on the ability of the enzyme to catalyze the desired reaction. Another way to explore the combinatorial space without exhaustive screening of the entire combinatorial space is to generate library pathways, but it may produce a nonoptimal solution or a dead end.^42^ By contrast, this work proposes to train a neural network to learn the combinatorial space from a few examples, and then use the trained network to scan the entire combinatorial space at high speed. Using NNs to solve the combinatorial libraries task has, thus, the potential to reduce the experimental (or computational) effort required to explore the combinatorial space in search of good enzyme mutants.

Feature injection has been used before in biological problems to either achieve higher performance^9,40^ or train with small data sets.^4,15,43^ And, the idea of using pretrained models to learn continuous vector embeddings to be used in combinatorial libraries has previously been attempted.^44^ In that early study, the method was unsuccessful and the authors note that protein design and protein classification (i.e., the task on which using the vector embeddings was successful) are two very distinct tasks and that the vector embeddings are only 100-*d*, which is not enough to represent a protein. In this study, the vector embeddings (sized [452,512]) come from a model pretrained on a data set of protein sequences 20 times larger.^9,45^ The method hereby presented was successful in increasing the accuracy of the network upon injection. The idea was that the trained embeddings (***h***_*i*_) contain information about the query sequence (***s***_*i*_) in the context of all protein sequences seen during training (***S*** = {***s***_1_,***s***_2_, ...,***s***_∼10 000_}). For example, mutation to Proline of a position within an α helix would prevent the continuation of the α helix, and decrease the ability of the LSTM model to predict the next residue in the sequence. The embeddings coming from this mutant sequence can be interpreted by the network to improve binding energy predictions (Figure S8).

In practice, the use-cases of the presented strategy are limited. The obvious application would be in exploring the entire combinatorial space to find the top-scoring variants (Figure 5C). However, the main problem is that the Rosetta-generated binding energies are never perfect, and the discovered variants may not necessarily work out in wet-lab experiments. The binding energies could be generated by more sophisticated or accurate methods (e.g., molecular dynamics simulations or experimental enzymatic activities) with the increased cost (computational or in lab equipment) in generating the training data set. Another possibility would be to train the model with a massive data set generated by a computationally inexpensive methodology (e.g., Rosetta) and later retrain it on the smaller but more accurate data set. Another application could be in exploring and comparing the entire combinatorial spaces of seemingly related enzymes (for example the ω-TA from *Vibrio fluvialis* and *Chromobacterium violaceum*). Beyond potential use cases, the success of the presented methodology in evaluating unseen mutants by a neural network unaware of the identity of the ligand or any structural information also hints that the task may not be as complex as initially thought.

## Conclusions

The proposed deep learning strategy for predicting the binding energy of enzyme variants with the ligand of interest achieved a high accuracy after training with a data set sized a miniscule fraction of the total combinatorial space. The methodology leverages the high combinatory power of neural networks to quickly learn patterns for combining mutants from a few examples. The naked GCN module achieved high accuracy when the number of mutation hotspots was small, but the accuracy decreased in data sets with a larger number of mutation hotspots. The combined LM-GCN module enhanced the predictions of the GCN module by injecting feature vectors generated by a pretrained LM module.

## Data Availability

Code is available at: https://github.com/crp-mol/super-HTS.

## Supporting Information Available

Additional figures of the structures of the ligands, protein complexes, training and testing procedures, extrapolation, and signal degradation (PDF)

## Abbreviations

GCN: graph convolutional network
ConvNet: convolutional neural network
DL: deep learning
MD: molecular dynamics
ω-TA: ω-transaminase
*Vf*-TA: ω-transaminase from *Vibrio fluvialis*
LM: language model
LSTM: long short-term memory
CNN: convolutional neural network
NN: neural network
FFT: fast Fourier transform
MSE: mean squared error
