# Supplementary material for: Super High-Throughput Screening of Enzyme Variants by Spectral Graph Convolutional Neural Networks
Source: J Chem Theory Comput. 2023 Mar 24;19(14):4668–77. doi: 10.1021/acs.jctc.2c01227 (PMC10373491; doi:10.1021/acs.jctc.2c01227)
Supplement: Supplementary file 1 — ct2c01227_si_001.pdf [file ct2c01227_si_001.pdf]

Supporting Information

Super High-Throughput Screening of Enzyme Variants by Spectral Graph Convolutional Neural Networks

Carlos Ramírez-Palacios[1] and Siewert J. Marrink[1]*

[1] Molecular Dynamics, Groningen Biomolecular Sciences and Biotechnology Institute (GBB), University of Groningen, Nijenborgh 7, 9747 AG Groningen, Groningen, The Netherlands

* Corresponding author
Groningen Biomolecular Sciences and Biotechnology Institute
University of Groningen
Nijenborgh 7
9747 AG Groningen
The Netherlands
E-mail: s.j.marrink@rug.nl

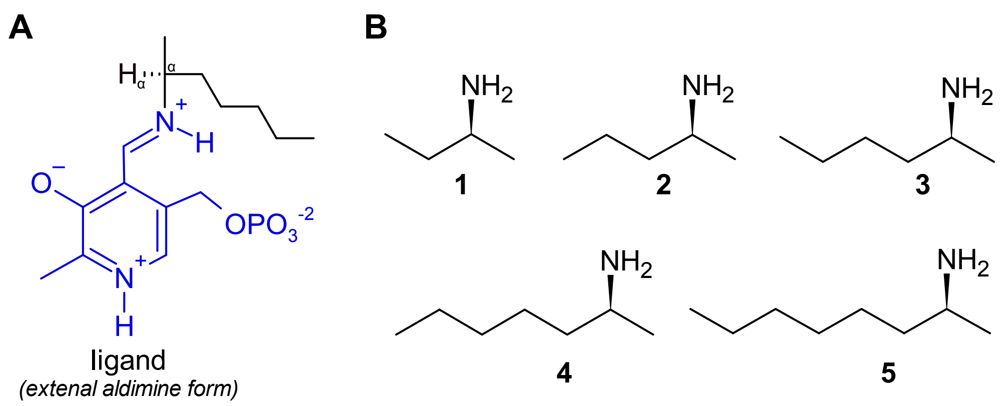

Figure S1. Chemical structure of the ligands used in this study. **A)** The main ligand (**E4**) is the external aldimine form of (2*S*)-heptan-2-amine. **B)** Additional compounds. In all cases, the ligand docked into the binding site of *Vf*-TA was the external aldimine form of the (*S*)-amine.

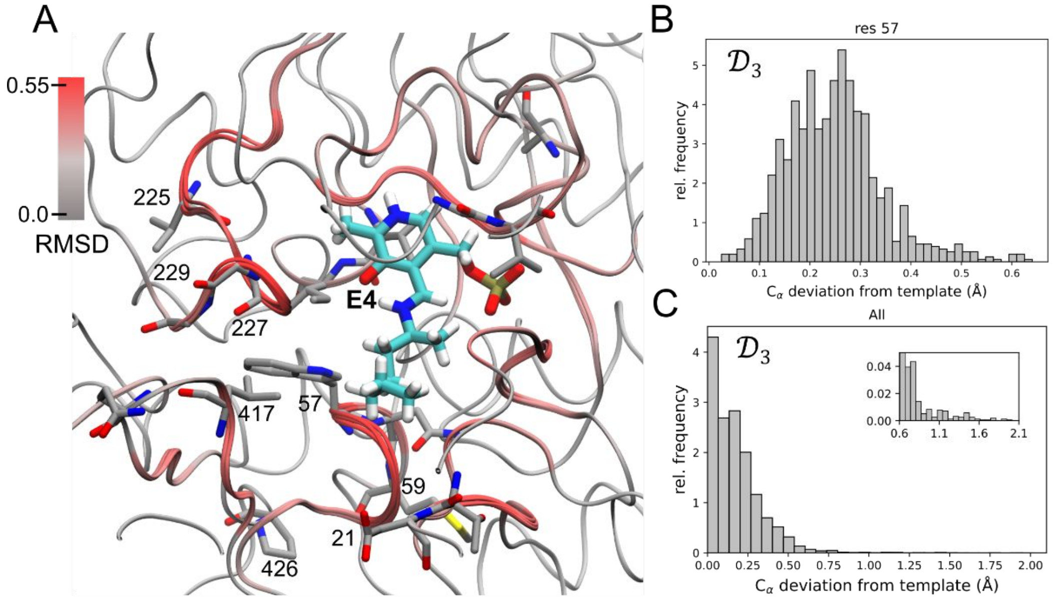

Figure S2. Mutations do not induce large conformational changes (given by the backbone C_α atoms), and therefore it is justified to consider the edge attributes as constant. **A)** Overlayed binding site structures of 1000 random Rosetta designs (dataset $\mathcal{D}_3$) colored by their RMSD deviation. The C_α atoms of the residues near the binding site (nodes) are not significantly moved by Rosetta, and therefore the graph edges representing the pairwise distances between residues stay constant. **B)** Histogram showing the deviation (d) of the $[x_i, y_i, z_i]$ coordinates of the C_α of residue 57 with respect to the C_α coordinates of the crystal structure $[x_0, y_0, z_0]$, $d = \sqrt{(x_i - x_0)^2 + (y_i - y_0)^2 + (z_i - z_0)^2}$. The histogram includes the deviations of 1000 Rosetta designs ($\mathcal{D}_3$), $1 \leq i \leq 1000$. The histogram of residue 57 is shown because it is one of the residues that moves the most (*red* in panel A), but its average deviation of 0.23 Å would hardly alter the graph representation of any of the mutants. **C)** Histogram showing the deviations of all residues near the binding site for the 1000 mutants (including residue 57). Most of the deviations fall below 0.5 Å, and only in very rare cases go above 1.0 Å. Again, the edge attributes of these mutants do not differ from the edge attributes taken from the crystal structure, and can thus safely be considered constant.

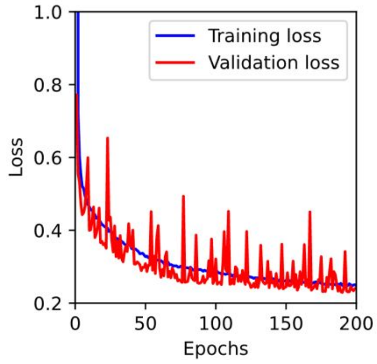

Figure S3. Training and validation loss over the 200 epochs ($\mathcal{D}_1$). There is no overfitting.

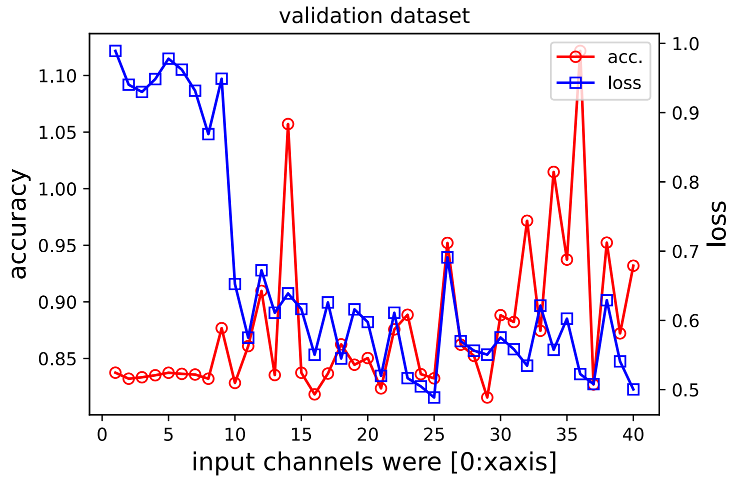

Figure S4. Effect the range of input channels has on the validation loss and accuracy of the trained model ($\mathcal{D}_1$). The features taken from the AAindex to represent every amino acid were range [0, x-axis], where x-axis is the position shown in the x-axis. It is shown that below 10 features, the neural network does not know enough about the residues to build a good model.

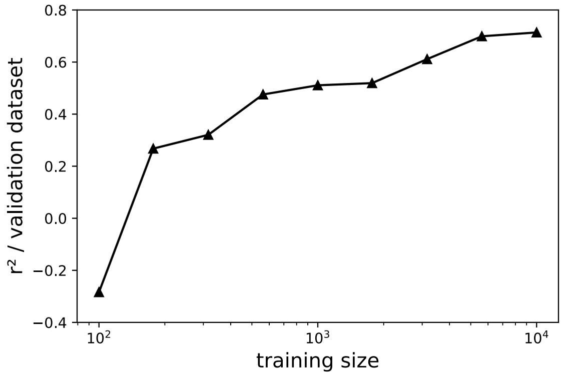

Figure S5. Effect of the size of the dataset on the performance (r^2) of the trained model ($\mathcal{D}_1$).

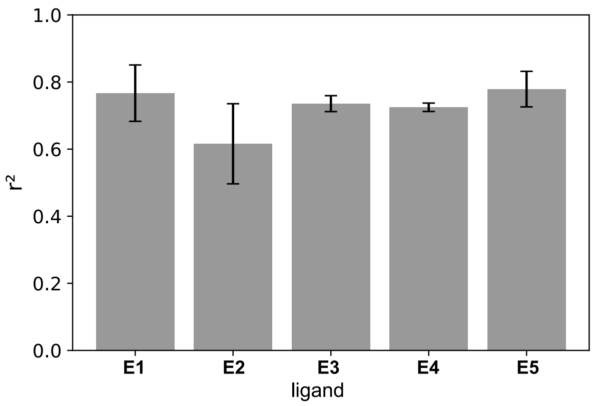

Figure S6. Correlation of y_i vs $\hat{y}_i$ of the GCN model trained and evaluated on five ligands ($N_{hot} = 4$).

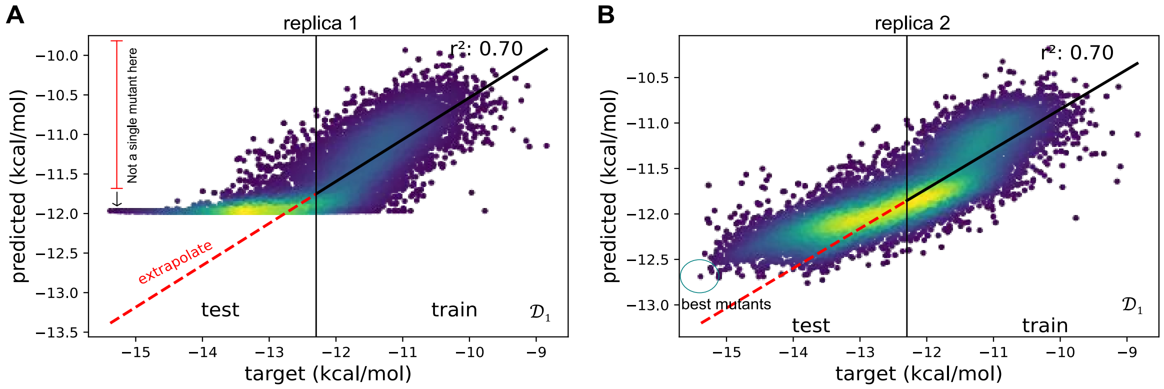

Figure S7. KDE scatter plot showing that a model trained on a subset of mutants (mutants with scores > -12.3 kcal/mol, labelled as *train*) might not always be able to extrapolate to mutants with much better scores (i.e., scores < -12.3 kcal/mol; labelled as *test*). The two panels are two replicas of the same model ran with different seeds. **A)** In this replica, the trained model is still able to score the best mutants (e.g., mutants with $x < -15.0$) with the best scores it can give them (i.e., ~ -12.0 kcal/mol) but it cannot give scores beyond said value. The model could have ranked the mutants of the *test* dataset with scores anywhere between -10.0 to -12.0 kcal/mol, yet it chose to rank most of them as -12.0 kcal/mol, which is the minimum score it learned from training. **B)** In this replica, the model does give scores to mutants in the *test* dataset that go beyond the boundaries of the *train* dataset. The best mutants ($x < -15.0$) were indeed identified as having top scores by the NN. The absolute x-axis values do not match the y-axis values in the *test* dataset but the trend is still present, and most importantly for protein engineering campaigns the model identifies the top candidates for further screening even in this situation.

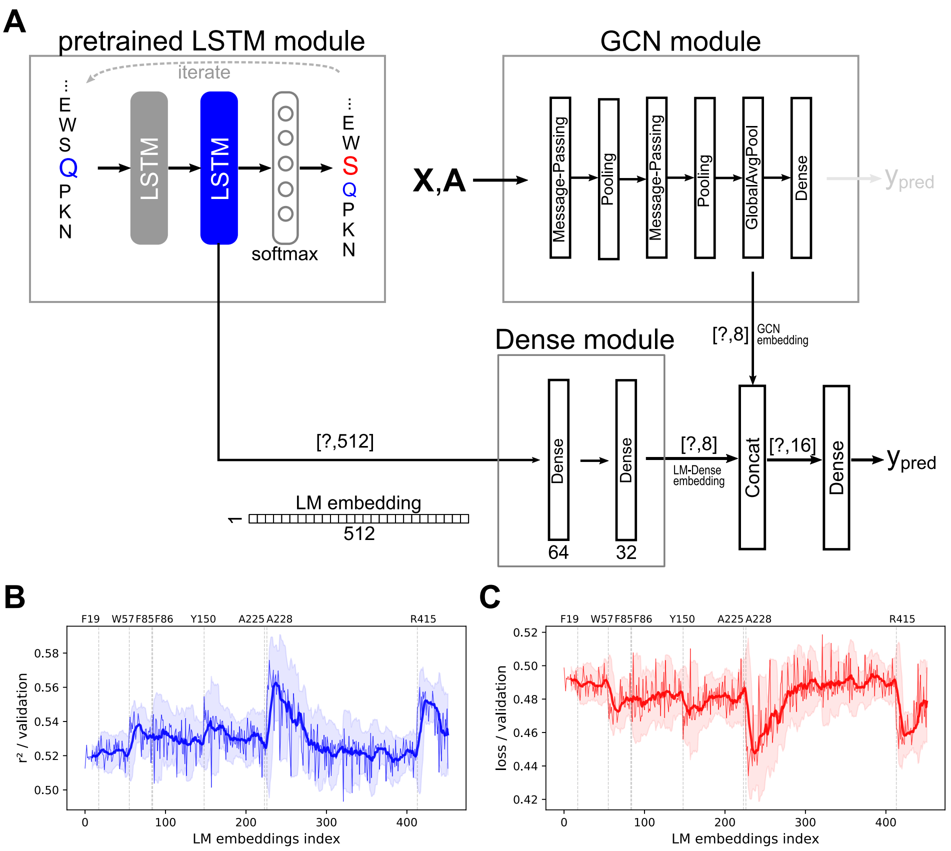

Figure S8. Performance of the trained LM-GCN model when using different LM embeddings ($\mathcal{D}_3$). **A)** LM-GCN model. **B)** r^2, **C)** Loss. The LM-GCN model performs better when fed the embeddings coming from right after the mutable positions (19, 57, 85, 86, 150, 225, 228, 415). After a while, the signal degrades and the performance decreases.
